# Over 30% efficiency bifacial 4-terminal perovskite-heterojunction silicon tandem solar cells with spectral albedo

**DOI:** 10.1038/s41598-021-94848-4

**Published:** 2021-07-30

**Authors:** Sangho Kim, Thanh Thuy Trinh, Jinjoo Park, Duy Phong Pham, Sunhwa Lee, Huy Binh Do, Nam Nguyen Dang, Vinh-Ai Dao, Joondong Kim, Junsin Yi

**Affiliations:** 1 Photoelectric and Energy Device Application Lab. (PEDAL), Multidisciplinary Core Institute for Future Energies (MCIFE), Incheon National University, Incheon, 22012 Republic of Korea; 2 Department of Energy Science, Sungkyunkwan Univeristy, 2066 Seobu-ru, Suwon-si, Gyeonggy-do 16419 Republic of Korea; 3 Department of Physics, International University, Block 6. Linh Trung Ward, Thu Duc District, Ho Chi Minh City, 720400 Vietnam; 4 Vietnam National University, Ho Chi Minh City, Vietnam; 5 Division of Energy and Optical Technology Convergence, Major of Energy Convergence Engineering, Cheongju University, 298, Daeseong-ro, Chungcheongbuk-do, Cheongwon-gu, Cheongju-si, 28503 Korea; 6 Division of Computational Physics, Institute for Computational Science, Ton Duc Thang University, Ho Chi Minh City, Vietnam; 7 Faculty of Electrical and Electronics Engineering, Ton Duc Thang University, Ho Chi Minh City, Vietnam; 8 Departement of Electrical and Computer Engineering, Sungkyunkwan University (SKKU), Seoul, 16419 Republic of Korea; 9 Ho Chi Minh City University of Technology and Education, 01 Vo Van Ngan, Thu Duc District, Ho Chi Minh City, 700000 Vietnam; 10 Future Materials and Devices Lab. (FM&D), Institute of Fundamental and Applied Sciences, Duy Tan University, Ho Chi Minh City, 700000 Vietnam; 11 Faculty of Electrical-Electronic Engineering, Duy Tan University, Da Nang, 550000 Vietnam; 12 Department of Electrical Engineering, Incheon National University, Incheon, 22012 Republic of Korea; 13 College of Information and Communication Engineering, Sungkyunkwan University (SKKU), Suwon, 16419 Republic of Korea

**Keywords:** Energy science and technology, Materials science

## Abstract

We developed and designed a bifacial four-terminal perovskite (PVK)/crystalline silicon (c-Si) heterojunction (HJ) tandem solar cell configuration albedo reflection in which the c-Si HJ bottom sub-cell absorbs the solar spectrum from both the front and rear sides (reflected light from the background such as green grass, white sand, red brick, roofing shingle, snow, etc.). Using the albedo reflection and the subsequent short-circuit current density, the conversion efficiency of the PVK-filtered c-Si HJ bottom sub-cell was improved regardless of the PVK top sub-cell properties. This approach achieved a conversion efficiency exceeding 30%, which is higher than those of both the top and bottom sub-cells. Notably, this efficiency is also greater than the Schockley–Quiesser limit of the c-Si solar cell (approximately 29.43%). The proposed approach has the potential to lower industrial solar cell production costs in the near future.

## Introduction

The highest recorded efficiency of a practical single-junction silicon solar cell is 26.7%, obtained under an interdigitated back contact (IBC) silicon heterojunction (HJ) configuration. The cell was developed by Kaneka in Japan^1^. However, this recorded efficiency remains lower than the theoretical efficiency limit of a single-junction silicon solar cell (i.e., 29.43%)^2^. The discrepancy between the practical and theoretical efficiencies can be attributed to thermalization and transmission loss^3–5^. To overcome this drawback, tandem solar cells consisting of multijunction configurations with various optical band gaps to harvest the entire solar spectrum range were developed following extensive investigations^3^. Both inorganic-inorganic and organic–inorganic materials have been examined to create tandem architectures^6–13^.

Tandem configurations can be roughly grouped into three categories, namely spectral splitting, four-terminal (mechanically stacked), and two-terminal (monolithically integrated)^14,15^. Among these, the optical splitting architecture showed an efficiency of approximately 28%^16^. However, the device configuration is complicated such that the device cannot be feasibly produced in bulk. In a monolithically integrated configuration, top and bottom sub-cells are electrically coupled in series; hence, the short-circuit current density (J_sc_) of the tandem device is limited by J_sc_ of the lowest sub-cell. Thus, the tandem power conversion efficiency (PCE) is constrained^7^. This implies that current matching between the top and bottom sub-cells must be strictly fulfilled and form the initial challenge concerning the fabrication of two-terminal tandem solar cells^10^. Another challenge concerns the high-quality deposition of the top sub-cell on the textured surface of the bottom silicon sub-cell^16^.

Alternatively, in the four-terminal tandem architecture, the top and bottom sub-cells are optically—but not electrically—coupled. The requirement for perfect current matching is thus negligible. The top and bottom sub-cells are independently fabricated; therefore, the high quality of the top sub-cell can be easily recreated on a glass substrate due to its smooth surface^8^. Some effort has been expended to create the four-terminal configuration in tandem devices. Quiros et al. optimized single-junction perovskite (PVK) solar cells via nanostructure transparent electrodes using a silver nanowire. The obtained efficiency was 26.7% and 25.2% for perovskite-passivated emitter rear locally diffused (PERL) and perovskite-IBC tandem devices, respectively^17^. Using the same device configuration, Wang et al. reported a conversion efficiency of 27.0% based on a perovskite-heterojunction with an intrinsic thin-layer (HIT) configuration. This was achieved by employing a MoO_3_/Au/MoO_3_ multilayer for a transparent electrode of the perovskite top sub-cell^14^. A similar architecture employing C_60_ for the electrode selective contact and a SnO_x_/ZTO/ITO transparent electrode for the Perovskite top sub-cell was created using a perovskite-silicon tunnel oxide passivated contact (TOPCon) tandem configuration (with a PCE of 26.7%)^18^. Another attempt to achieve the same device configuration involved copper indium gallium selenide (CIGS) instead of crystalline silicon (c-Si) based material for the bottom sub-cell. The obtained conversion efficiency of ~ 5.9% was lower than that of the perovskite-Si-based HJ tandem solar cells^8^. By employing a charge transfer–induced 2,9-dimehtyl-4,7-diphenyl-1,10-phenanthroline (BCP):Ag complex between a C_60_ electron-transport layer and sputtered indium-zinc oxide top electrode to reduce sputtering damage for underlayer, the efficiency of the semi-transparent perovskite solar cells (PSCs) was improved from 16.14 to 18.19%. An efficiency of 27.59% was then obtained for the four-terminal PSCs/TOPCon configuration^19^. The four-terminal with bifacial PSCs–metal wrap through silicon hetero-junction configuration was also reported; using this configuration, a device conversion efficiency of 30.5% was reached^20^.

Prior studies indicate that the standard-alone Si-based HJ solar cell always exhibits high efficiency (> 20%) in defiance of the cell structure. Nevertheless, the PCE of the PVK-filtered Si-based HJ bottom sub-cell showed an efficiency of less than 10%. This occurred because a short wavelength range was yielded by the perovskite top sub-cell, following which the J_sc_ of the bottom sub-cell, and, accordingly, the PCE were reduced. Studies have attempted to improve J_sc_ of the bottom sub-cell via minimized absorption using the perovskite sub-cell, which can be accomplished using high-band-gap perovskite material or highly transparent electrodes. Therefore, to achieve higher efficiency in tandem solar cells, the top and bottom sub-cells must fulfill solar spectra with respect to their optical band gaps. To meet this requirement, a bifacial four-terminal perovskite-HIT tandem solar cell with spectral Albedo was investigated in this study for the first time. This design configuration is advantageous thought its ability to improve J_sc_ of the bottom sub-cell via spectral albedo, in defiance of the top sub-cell properties and without raising costs compared with mono-facial tandem cells.

## Results and discussion

### Evaluating top sub-cell candidates for the bifacial four-terminal tandem solar cell

**Figure 1:**
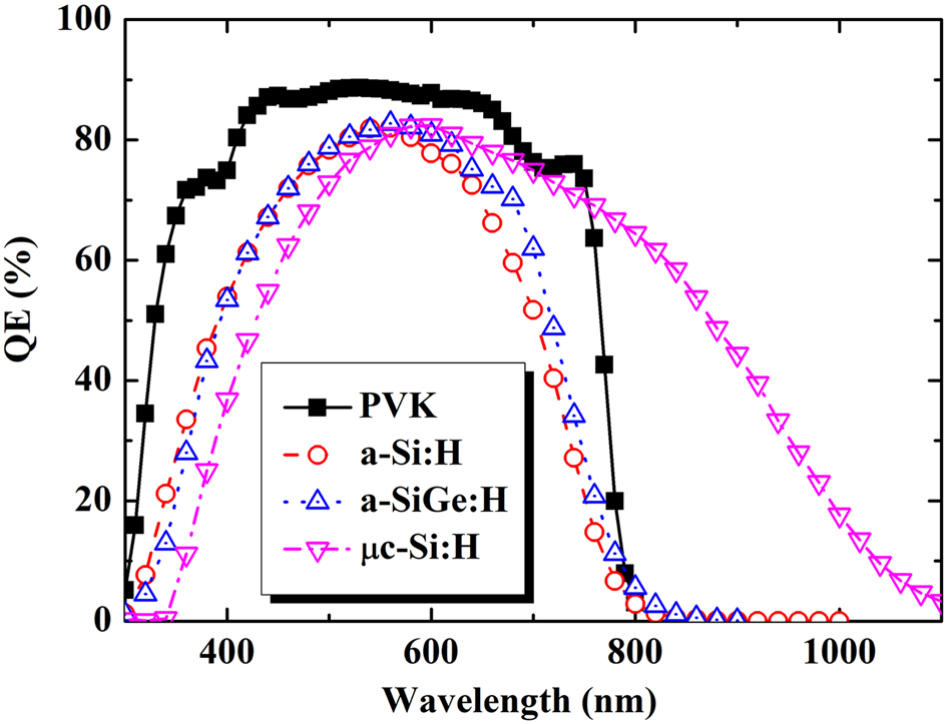
Measured QE spectra of the various top sub-cell candidates. The black, red, blue, and magenta lines depict the QE of the PVK, a-Si:H, a-SiGe:H, and μc-Si:H thin film solar cell, respectively.

The preceding discussion suggests that the PCEs of individual sub-cells must be as high as possible, without presenting sub-cell current density matching, to obtain the highest PCE of the bifacial four-terminal tandem device. Recently, organic and inorganic materials, such as hydrogenated amorphous silicon (a-Si:H), hydrogenated amorphous silicon germanium (a-SiGe:H), hydrogenated microcrystalline silicon (μc-Si:H), and PVK^3,6–10,12–18^, have been employed for the top sub-cell. To classify the appropriate top sub-cell candidates for tandem device application, different single-junction thin film solar cells were fabricated and characterized. Figure 1 presents the quantum efficiencies (QEs) for different types of top sub-cell candidates. QE curves can be roughly divided into two zones, namely, the short-wavelength region (< 600 nm) and long-wavelength region (> 600 nm). The QE behavior of the a-Si:H and a-SiGe:H thin-film solar cells (TFSs) shows negligible differences in the wavelength range of 300 to ~ 550 nm, whereas that of a-SiGe:H TFS shows an improvement in the wavelength range of ~ 550 to ~ 800 nm when compared to the a-Si:H TFS. Such an improvement can be attributed to the improved light absorption in the long-wavelength range for the case of a-SiGe:H with a low optical band gap, as reported previously^3^. Figure 1 also exhibits the QE behavior of the μc-Si:H TFS. The result thereof confirmed the enhanced infrared absorption of the μc-Si:H TFS compared with the amorphous silicon TFS. It is interesting to note that the perovskite top sub-cell’s QE indicates a significant enhancement in the all-wavelength zone of interest compared with semitransparent amorphous silicon TFS. The semitransparent perovskite sub-cell, on the other hand, shows substantial improvement in the wavelength range of 300 nm to ~ 800 nm. However, the cut-off of the perovskite’s QE occurs at a wavelength of 800 nm, whereas that of the μc-Si:H TFS is still available up to 1100 nm.

**Figure 2:**
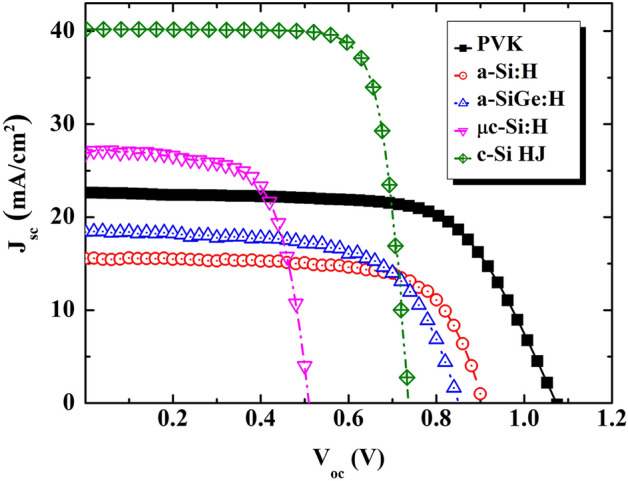
Illuminated current–voltage characteristics of the various top sub-cell candidates and standard-alone a-Si:H/c-Si heterojunction solar cell under 1 sun. The black, red, blue, magenta, and olive lines indicate the J-V curves for the PVK, a-Si:H, a-SiGe:H, μc-Si:H thin film solar cell and c-Si HJ solar cell, respectively.

Base on the experimental QE results, the integrated photocurrents were simulated and are presented in Table 1. The calculated results may be predicted. The highest integrated photocurrent is 26.76 mA/cm^2^ for the μc-Si:H solar cell, followed by that for the semitransparent perovskite (22.60 mA/cm^2^), a-SiGe:H (18.50 mA/cm^2^), and a-Si:H (15.65 mA/cm^2^) TFS. Therefore, the semitransparent μc-Si TSF and perovskite TSF are potential candidates for achieving high conversion efficiency in the top sub-cell of a four-terminal tandem device. In addition, the characteristics of the different semitransparent cells were examined and are presented in Fig. 2 and Table 1. These results are similar to those of the QEs. The μc-Si TSF has the highest J_sc_ of 26.81 mA/cm^2^, followed by perovskite TFS (J_sc_ of 22.66 mA/cm^2^), a-SiGe:H TFS (J_sc_ of 18.32 mA/cm^2^), and a-Si:H TFS (J_sc_ of 15.56 mA/cm^2^). However, the behavior of V_oc_ was found to be different than that of J_sc_. The highest V_oc_ was obtained for the perovskite TFS (1100 mV), followed by a-Si:H TFS (900 mV), a-SiGe:H TFS (850 mV), and μc-Si TFS (510 mV). Finally, the PCE gathers all the characterizations of the device performance. This information is also presented in Table 1. Here, all amorphous silicon and microcrystalline silicon exhibited a PCE of ~ 10%. Notably, the semitransparent perovskite solar cell had a PCE of up to 17.1%. Thus, our analysis indicates that the fabricated semitransparent perovskite thin-film solar cell is potentially the top candidate for the top sub-cell in a four-terminal tandem solar cell, in defiance of the highest J_sc_ from the μc-Si:H thin-film solar cell.

**Table 1:** Calculated J_sc_ and performance metrics of various top sub-cell candidates.

	J_sc_ (mA cm^2^)		V_oc_ (V)	FF (%)	Efficiency (%)
	Cal.	Meas.			
PVK	22.60	22.66	1.10	68.60	17.10
a-Si:H	15.65	15.56	0.90	70.40	9.86
a-SiGe:H	18.50	18.32	0.85	64.20	10.00
μc-Si:H	26.76	26.81	0.51	68.22	9.33

### Bifacial four-terminal tandem solar cell fabrication and characterizations

**Figure 3:**
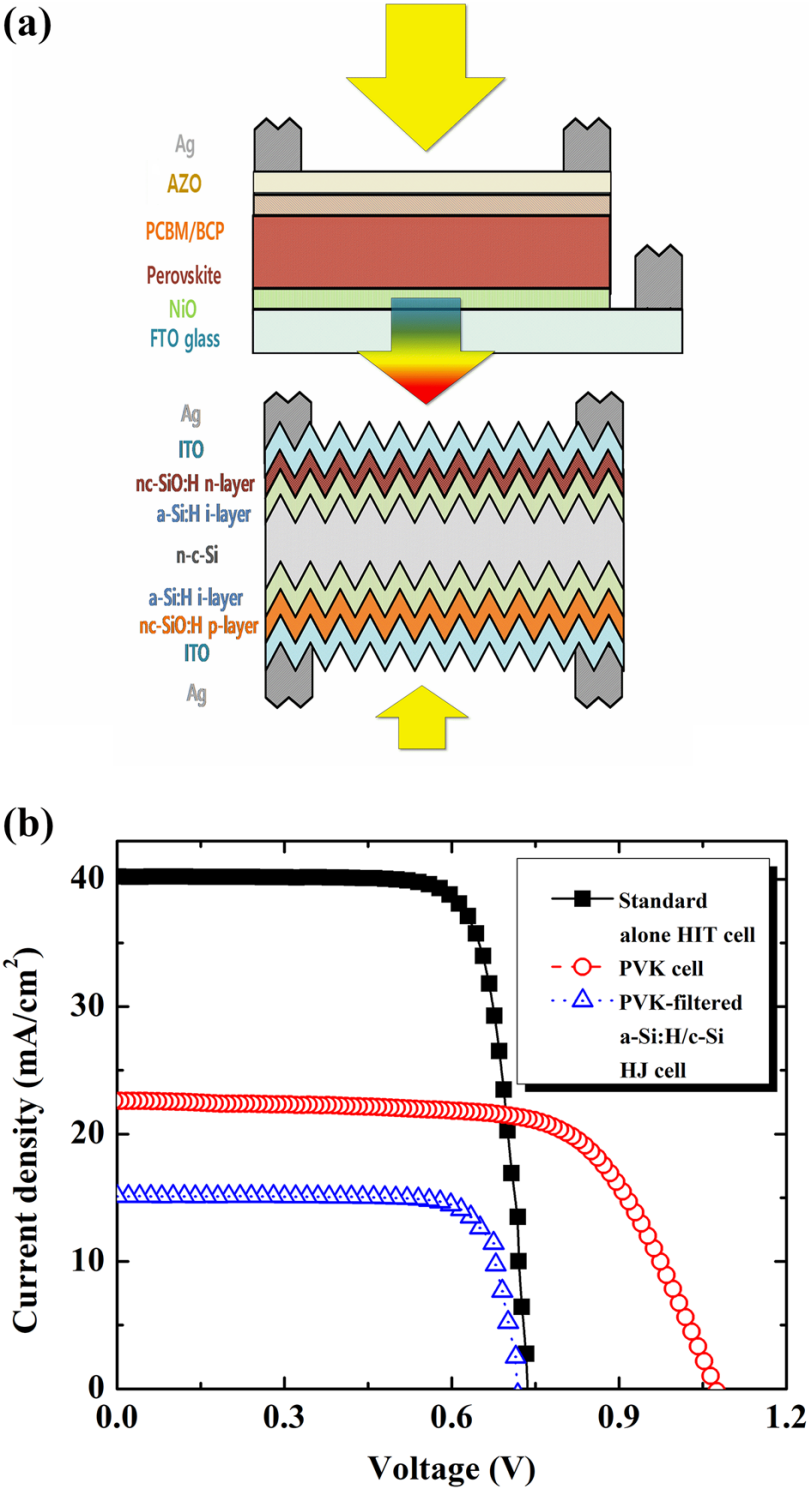
(**a**) Schematic of the bifacial 4-terminal PVK/c-Si HJ tandem solar cell with albedo reflection. (**b**) Illuminated current–voltage curves under 1 sun of the standard-alone c-Si HJ solar cell (black color), PVK filtered c-Si HJ solar cell (blue color) and the PVK solar cell (red color).

The four-terminal tandem solar cell structure was constructed, as shown in Fig. 3a. The semitransparent perovskite serves as the top sub-cell, that is, a solar filter for the a-Si:H/c-Si bottom sub-cell. Figure 3b shows the performance of the bifacial four-terminal tandem device under 1 sun illumination on the front side. The characteristics of the standard-alone a-Si:H/c-Si HJ solar cell without a PVK filter were measured and are presented in Fig. 3b. The obtained results indicate that the performance of the PVK remains unchanged. By contrast, several characteristics of the PVK-filtered a-Si:H/c-Si HJ solar cell showed a significant decrease, particularly J_sc_ (from ~ 40.00 mA/cm^2^ to ~ 15.15 mA/cm^2^). Hence, the conversion efficiency decreased significantly, from 23.35 to 8.68%.

**Figure 4:**
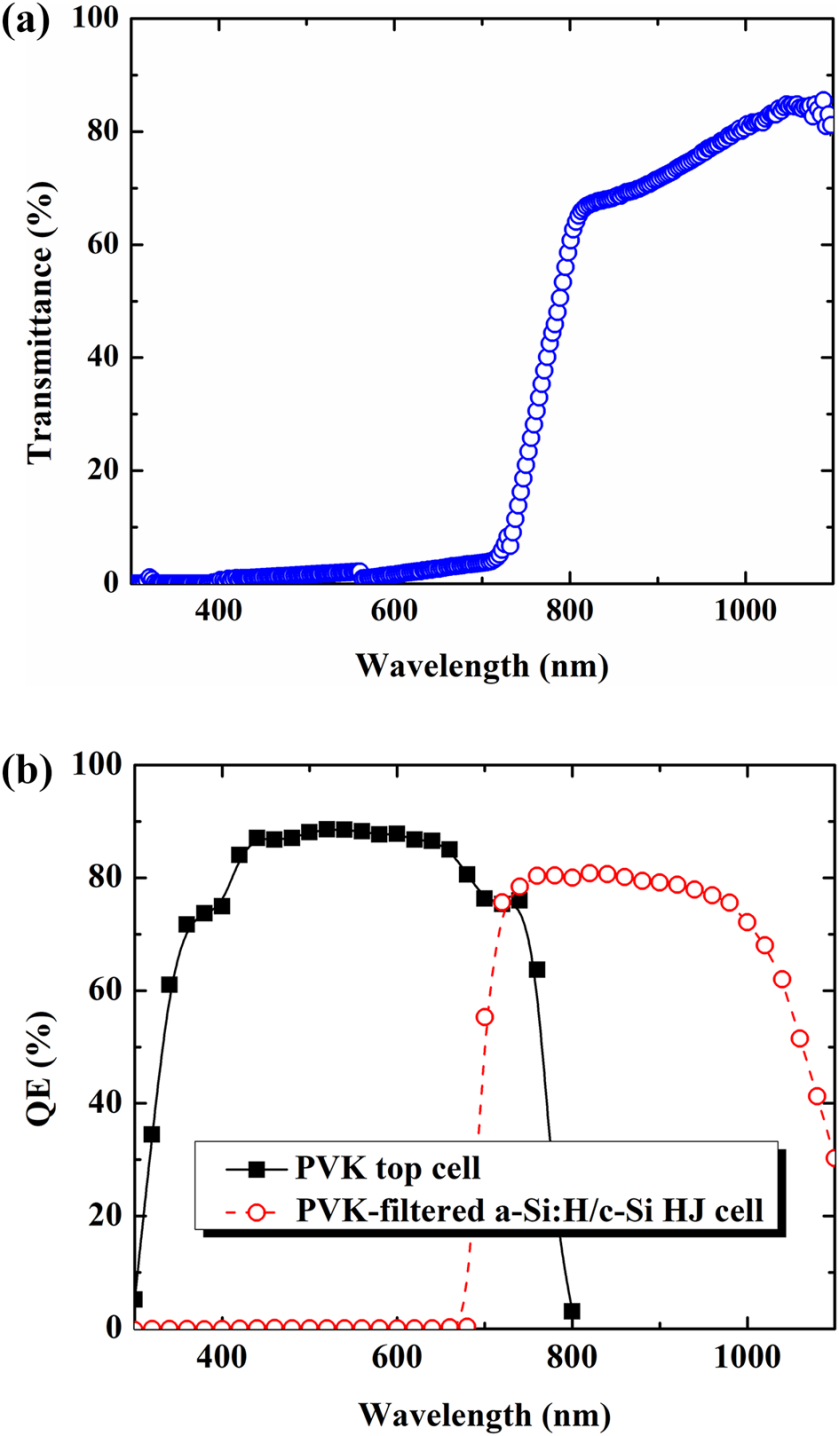
(**a**) Transmittance through perovskite top sub-cell solar cell. (**b**) Quantum efficiency of the PVK top sub-cell and of the PVK-filtered c-Si HJ solar cell.

To elucidate the main reason for J_sc_ reduction of the PVK-filtered HIT sub-cell, the transmittance of the PVK cell was investigated, as depicted in Fig. 4a. The PVK cells absorbed all solar spectra in the short-wavelength range. This implies that the solar spectrum that reaches the bottom sub-cell is lower than that without PVK filtering, leading to decreased electron–hole pair generation, which can be detrimental to V_oc_ and particularly J_sc_. The result of a low J_sc_ for a PVK-filtered HIT bottom sub-cell can be clearly understood upon analyzing the QE results. Figure 4b shows the QE of a PVK sub-cell and PVK-filtered HIT sub-cell. Based on the QE results, the calculated photocurrents are 21.50 and 16.22 mA/cm^2^ for the PVK top sub-cell and PVK-filtered HIT bottom sub-cell, respectively. These values are consistent with the experimental results. A conversion efficiency (sum of the conversion efficiencies of the PVK cell and the PVK-filtered cell) of 25.78%, which is higher than that of each independent sub-cell, was achieved for the four-terminal perovskite-HIT configuration.

**Figure 5:**
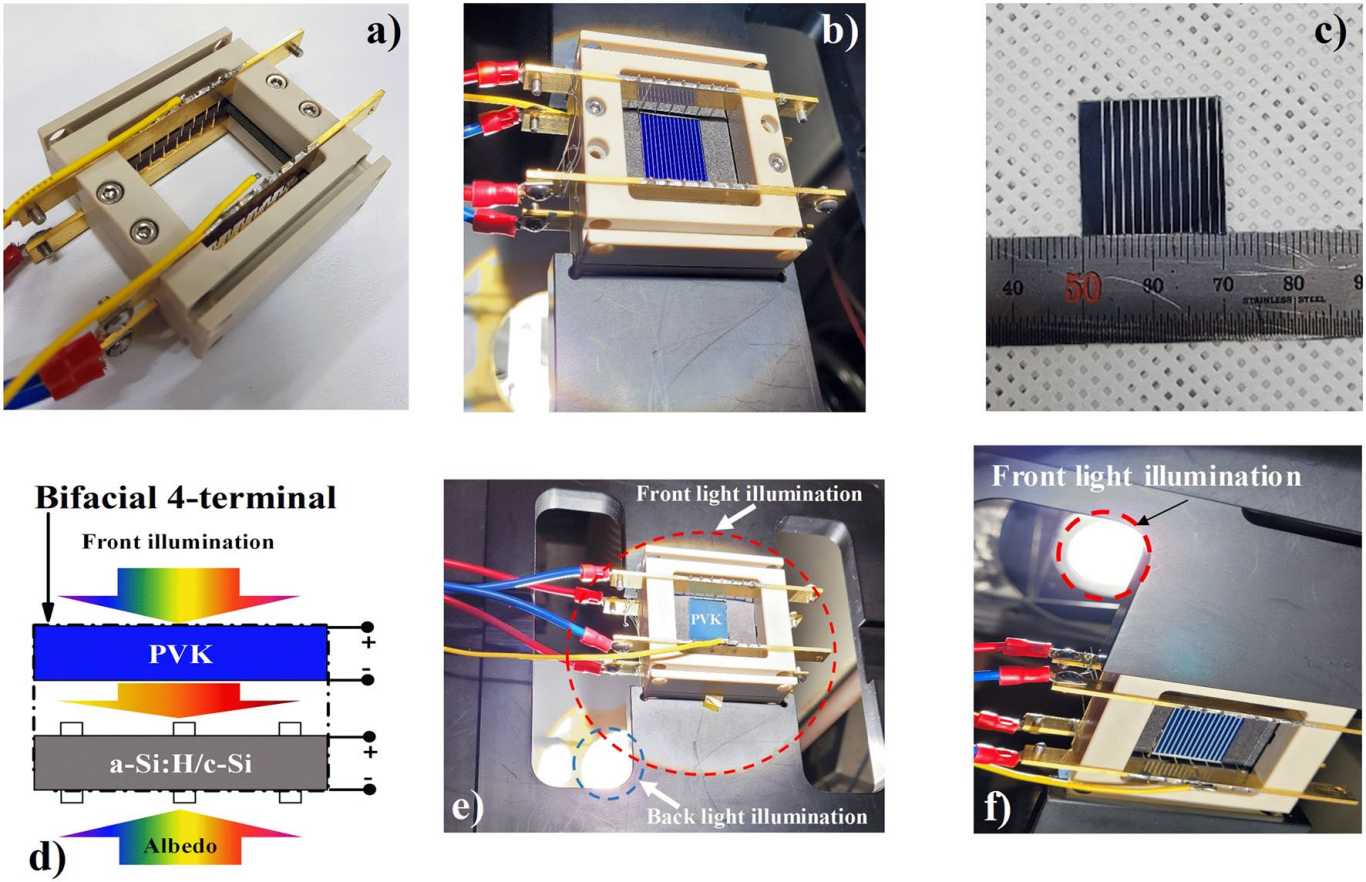
(**a**) A photo image of a solar cell Zig for bifacial 4-terminal tandem solar cell measurement. (**b**) The solar cell Zig with an a-Si:H/c-Si HJ solar cell. (**c**) A real image of the semitransparent PVK solar cell. (**d**) A configuration of the front and albedo light source; the distance between the cell’s holder and the albedo light source can be adjusted. (**e**) The solar cell Zig with PVK/c-Si HJ tandem solar cell and bi-directional light irradiation turned on. (**f**) The real image from back-side view of the bifacial 4-terminal PVK/c-Si HJ tandem solar cell with light source turned on.

**Figure 6:**
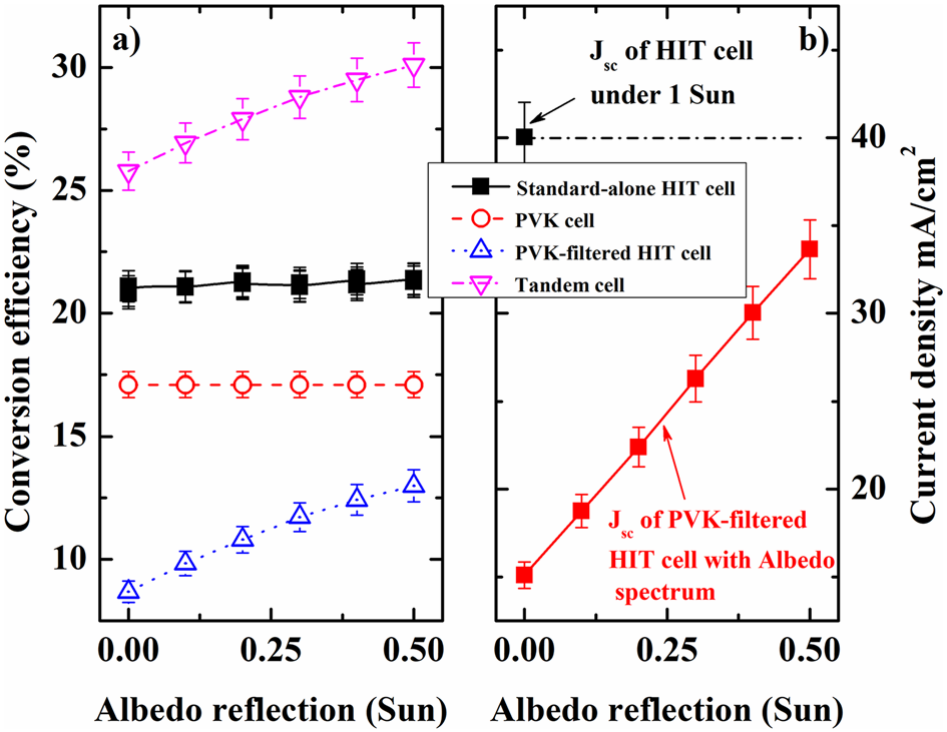
(**a**) The conversion efficiency of the PVK top sub-cell (red color), standard-alone c-Si HJ solar cell (black color), PVK-filtered c-Si HJ solar cell (blue color), and a bifacial 4-terminal PVK/c-Si HJ tandem solar cell (pink color) as a function of albedo reflection. (**b**) *J*_*sc*_ of the standard-alone c-Si HJ solar cell under 1 sun (black color) and the *J*_*sc*_ of the PVK-filtered c-Si HJ solar cell bottom sub-cell as a function of the albedo spectrum (red color).

**Figure 7:**
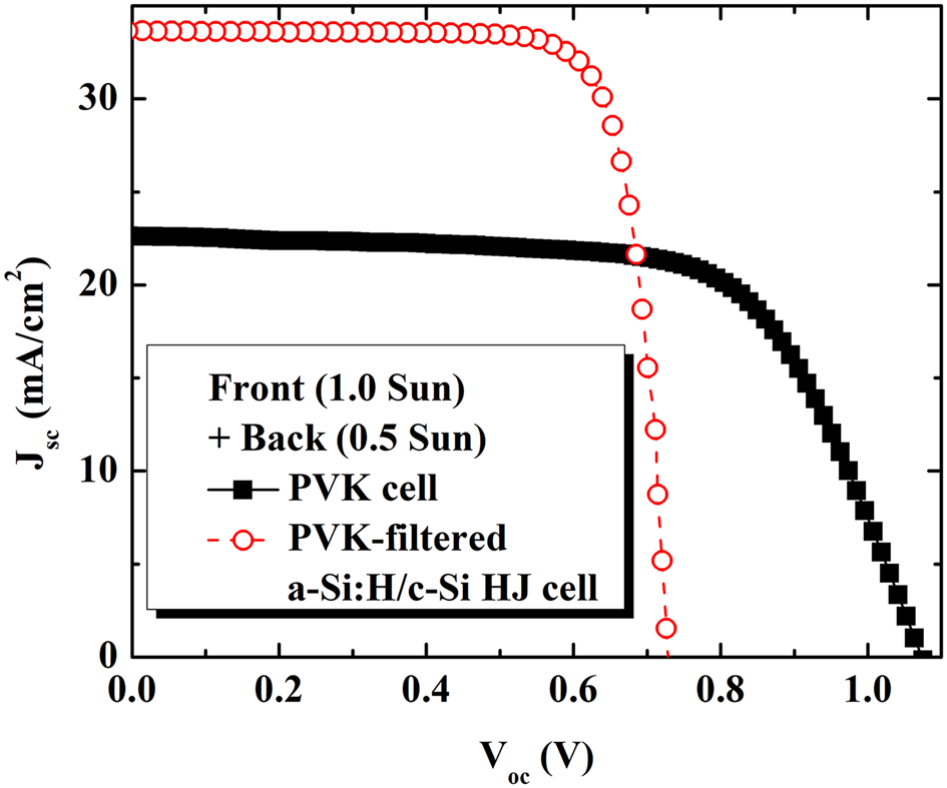
Representative J–V curves of the PVK top sub-cell and PVK-filtered a-Si:H/c-Si HJ bottom sub-cell in a bifacial 4-terminal PVK/c-Si HJ tandem solar cell under 1.0 sun (front-side) and 0.5 sun (back-side).

Thus, the a-Si:H/c-Si HJ bottom sub-cell performance remained significantly below that under the standard operation condition (AM 1.5 G or 1 Sun) (J_sc_ of ~ 15.15 mA/cm^2^ of the PVK-filtered HIT cell is significantly below the state-of-the-art value of the standard-alone HIT cell with a J_sc_ of ~ 40 mA/cm^2^), owing to the PVK-filter on the top, as seen in Fig. 4b. To push the PVK-filtered a-Si:H/c-Si HJ bottom sub-cell works close to 1 sun condition or the state-of-the-art, causing a rise in the J_sc_ and conversion efficiency, the bifacial 4-terminal tandem design with spectral albedo was also promoted (Fig. 3a). Here, the albedo reflection intensity can be accurately adjusted in the range of 0–0.5 sun by altering the distance between the cell holder and illumination source, as seen in Fig. 5. A calibrator was used to accurately control the albedo reflection. The advantage of using the bifacial solar cell is its acquisition of the solar spectrum from both sides: direct solar spectrum from the front side and reflected solar spectrum from the background (green grass, white sand, red brick, roofing shingle, snow, etc.)^6^. Figure 6a shows the efficiencies of the bifacial standard-alone a-Si:H/c-Si HJ and the bifacial four-terminal perovskite/c-Si HJ tandem solar cell as a function of the albedo spectrum. Here, the front illumination was kept fixed at 1 sun. The conversion efficiency of the top PVK cell (red circle) remains nearly unchanged, whereas that of the bifacial standard-alone HIT cell (black square) showed slight improvement with the albedo reflection. The conversion efficiency of the PVK-filtered HIT bottom sub-cell (blue triangle) showed a significant enhancement from 8.68 to 12.99% when the spectral albedo increased from 0 to 0.5 sun (refer to the supporting information for details in Fig. S1, S2). Consequently, the conversion efficiency (sum of the efficiencies of the PVK and PVK-filtered HIT cells) of the bifacial four-terminal tandem cell (pink triangle) showed a significant improvement from 25.78 to 30.09%, apparent in Fig. 7 and Table 2. To date, this remains a comparatively high conversion efficiency value achieved in PVK/HIT tandem solar cells.

**Table 2:** Summary of photovoltaic parameters for semitransparent PVK, a-Si:H/c-Si HJ solar cell under 1.0 sun (front) and 0.5 sun (back) and for the summed 4-terminal bifacial PVK/c-Si tandem device.

Solar cell	V_oc_ (V)	J_sc_ (mA/cm^2^)	FF (%)	The normalized conversion efficiency (%)
PVK cell	1.100	22.66	68.60	17.10
PVK-filtered a-Si:H/c-Si HJ cell	0.728	33.64	79.52	12.99
Tandem solar cell	–	–	–	30.09

The current density of the PVK-filtered HIT sub-cell as a function of the albedo spectrum was measured, as seen in Fig. 6b. The J_sc_ of the standard-alone HIT cell (black square) under 1 sun is also presented for reference. As shown in Figs. 6b, 7 and Table 2, we observe a monotonous increase in the J_sc_ of the PVK-filtered HIT cell (red square, Fig. 6b) from ~ 15.15 mA/cm^2^ to ~ 33.64 mA/cm^2^ (Fig. 7 and Table 2) and in the albedo reflection from 0 sun to 0.5 sun. It is worth noting that the highest J_sc_ with an albedo reflection of 0.5 sun is still lower than that of the standard J_sc_ value of the HIT cell (~ 40 mA/cm^2^). It is suggested that a higher J_sc_ regarding the upper limits of the c-Si HJ solar cell of approximately 42 mA/cm^2^ can be achieved using albedo reflection for the bifacial four-terminal tandem cell. To achieve higher conversion efficiency, further investigations such as lesser plasma damage of underlying organic–inorganic layers^21^ or higher albedo light intensity are required.

### Bifacial 4-terminal PVK/HIT tandem solar cell modeling

**Figure 8:**
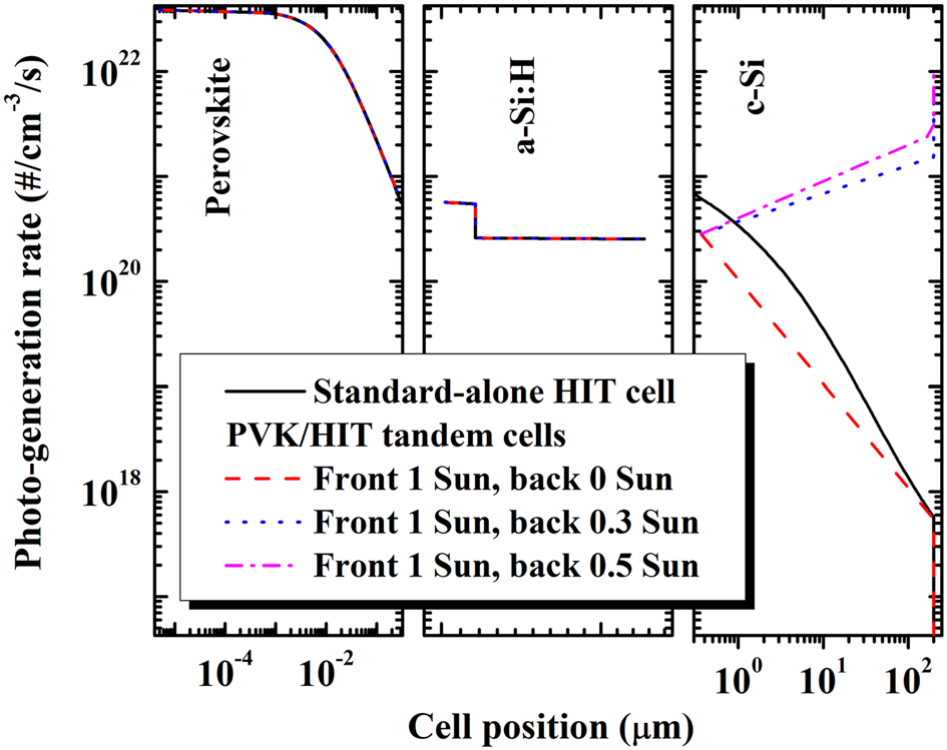
Plots of the photo-generated rate in a bifacial 4-terminal PVK/c-Si HJ tandem solar cell, with variation in the albedo spectrum, as a function of the cell position; here, the red, blue, and pink lines indicate albedo reflection at 0, 0.3, and 0.5 sun, respectively. The photo-generated rate of the standard c-Si HJ solar cell, as a function of the cell position, was also represented for reference (black line).

Simulated results were employed to examine the PVK/c-Si HJ tandem solar cell to elucidate the reason for the improvement in J_sc_ and, thereby, the conversion efficiency. Figure 8 shows the simulated photo-generated rate distribution, which was determined using AFORS-HET software, within the PVK/c-Si HJ tandem solar cell under three selected conditions: without spectra albedo (dashed red line), with spectra albedo of 0.3 sun (dotted blue line) and with spectra albedo of 0.5 sun (dash-dotted magenta line). For reference purposes, the simulated photo-generated rate distribution inside the a-Si:H/c-Si HJ solar cell without a PVK filter (solid black line) is plotted as a function of the cell position. We observed that the bottom sub-cell of the PVK/HIT tandem solar cell without albedo reflection shows fewer photo-generated electron–hole pairs in the entire device length than the standard-alone a-Si:H/c-Si heterojunction solar cell, a matter which deems detrimental to all parameters of the bottom sub-cell, as discussed earlier.

For PVK/c-Si HJ tandem solar cells, the photo-generated rate was kept unchanged through the PVK and emitter region (a-Si:H) of the bottom sub-cell, as opposed to the albedo reflection conditions. As a results, the albedo reflection did not contribute to the performance of the PVK cell, where we notice no change in the PCE of the PVK with variation in the albedo spectrum intensities. Using the albedo spectrum, the generated electron–hole pairs were improved remarkably along the bottom sub-cell position, particularly those close to the end of the cell. The higher the albedo reflection is, the higher the photo-generated rate will be.

In summary, a bifacial four-terminal PVK/c-Si HJ tandem solar cell with albedo reflection was proposed in this study. In a traditional four-terminal PVK/c-Si HJ configuration, the solar spectrum in the short-wavelength region is absorbed by the PVK top sub-cell, leaving the remaining spectrum to be absorbed by the c-Si HJ bottom sub-cell. However, not all photons corresponding to bottom sub-cell’s optical bandgap can be yielded. The J_sc_ (~ 15.15 mA/cm^2^) and consequent the conversion efficiency (8.68%) values for the bottom sub-cell are, therefore, far below the state-of-the-art values of the standard cell. Our approach employed a bifacial four-terminal PVK/c-Si HJ tandem structure with an albedo reflection, which increases the number of absorbed photons into the a-Si:H/c-Si HJ bottom sub-cell. As a result, J_sc_ and the conversion efficiency of the c-Si HJ bottom sub-cell improved from ~ 15.15 to 33.5 mA/cm^2^ and from 8.68 to 12.99%, respectively, when the albedo reflection increased from 0 to 0.5 sun. The excellent photon yielding of the a-Si:H/c-Si HJ sub-cell enabled a high J_sc_. We showed that the conversion efficiency of the PVK/HIT tandem solar cell was as high as 30.10%. These findings encourage further studies on the improvement of the conversion efficiency of four-terminal tandem solar cell using albedo reflection and/or low plasma damage of underlying organic–inorganic layers^21^.

## Methods

For the PVK solar cell fabrication, methylammonium-lead (ΙΙ) iodide (CH_3_NH_3_PbI_3_) was employed as the absorbent layer, which was positioned between a NiO layer and phenyl C_61_-butyric acid methyl ester (PCBM) material. The NiO and PCBM layers served as hole and electron transport materials, respectively. The final structure of the PVK solar cell is, thus, glass/fluorine-doped tin oxide (FTO)/NiO/absorber/PCBM/BCP/aluminum-doped zinc oxide (AZO)/Ag. FTO glasses were treated in ultrasonic bath with acetone, ethanol, and DI water, for 10 min each in sequences. An atomic layer deposition (ALD) system was employed to deposit the NiO hole transport layer at a temperature of 200 °C. Stoichiometric MAPbI_3_ solution was prepared by mixing DMF and DMSO solvents to prepare the perovskite precursor solution. The precursor solution, after being stirred overnight in an inert atmosphere glove box, was dropped on the NiO layer in accordance with the spin coating method, after which the layer was dried on a hot plate to form the perovskite absorber layer. The PCMB/BCP electron transport layer was then coated on the absorber layer according to the spin coating technique; AZO was grown at 100 °C by using the ALD system. Finally, the Ag electrode was developed using thermal evaporation with a shadow mask. The detailed fabrication process can be found in a previous publication by Seo et al.^22^.

For an a-Si:H/c-Si HJ solar cell fabrication, commercial Czochralski-grown n-type c-Si wafers 200 μm thick with an electrical resistivity of ~ 2.7 Ω·cm were used as the absorbent layer. Henceforth, the a-Si:H/c-Si HJ solar cell is termed the HJ solar cell. Passivated a-Si:H(i) layers were first deposited on both sides of the wafers. The nc-SiO:H(p) layer was then deposited on top of the a-Si:H(i) at one side as hole collector, whereas nc-SiO:H(n) layer was deposited on top of the a-Si:H(i) on the other side as the electron collector.

All a-Si:H layers were grown via plasma-enhanced chemical vapor deposition in a cluster system. For a bifacial configuration, 80 ± 5-nm-thick ITO was coated using a magnetron sputtered on top of the nc-SiO:H(p) and nc-SiO:H(n) layers in sequence. Finally, Ag finger grids were printed on top of the ITO layers via a low-temperature screen-printing method. The details of the growth conditions employed for all layers can be found in a previous report^23^. The final structure of the HJ solar cell is as follows: Ag(finger-grids)/ITO/nc-SiO:H(n)/a-Si:H(i)/c-Si(n)/a-Si:H(i)/nc-SiO:H(p)/ITO/Ag(finger grids).

To measure the current density–voltage characteristic of the bifacial four-terminal tandem solar cell, the bifacial solar cell Zig was designed for bi-directional investigation, as illustrated in Fig. 5a. Real photographs of a bifacial solar cell Zig with an a-Si:H/c-Si HJ solar cell are shown in Fig. 5b. Real photographs of semitransparent PVK solar cell and measurement schematics are shown in Fig. 5c,d, respectively. An actual photo images of the PVK/c-Si HJ tandem solar cell with bi-directional light irradiation are shown in Fig. 5e. Finally, a view from back-side of an actual photographs of the PVK/c-Si HJ solar cell with bi-directional light irradiation are shown in Fig. 5f. Here, we used two xenon lamps for the bi-directional investigation of PVK/c-Si HJ tandem solar cell characteristics. The light intensity of the first Xenon lamp (front side) was kept unchanged at 1 sun (a light intensity of 100 mW/cm^2^), whereas the light intensity of the Xenon lamp at the back side (spectral albedo) was adjusted from 0.0 to 0.5 sun by changing the distance between the back-side’s cell and the light source. For accurate controls of the light intensity, a calibrated reference cell (Newport, 91150-KG) was used. It is noted that in all measured setups, the temperature was maintained at 25 °C. The conversion efficiency of the bifacial four-terminal configuration cell can be determined by adding the conversion efficiency of the PVK cell and the PVK-filtered a-Si:H/c-Si HJ bottom sub-cell as follows:

1 $$ \eta_{Bifacial{-}4T} = \eta_{PVK {-} Top} + \eta_{{PVK{-}filtered \, c {-} Si \, HJ \, bottom,alb}} $$

Here the conversion efficiency of the PVK-filtered c-Si HJ bottom sub-cell, *η*_*PVK fileted c-Si HJ bottom, alb*_, is the normalized conversion efficiency (output power normalized to 1 sun illumination, that is, for 100 mW/cm^2^)^6^. The optical transmittance of the PVK cells was characterized using an ultraviolet–visible spectrophotometer in the wavelength range of 300–1100 nm (Sinco S-3100). The QE of each sub-cell was determined at 25 °C using a solar cell spectrum response/QE/incident photo-to-current efficiency measurement system G1218a (PV Measurements, Inc., Boulder, Co, USA). Automat FOR the Simulation of HETerostructure software (AFORS-HET 3.0.1) was utilized to characterize the photo-generation rate within the PVK-HJ tandem solar cells^24^.

## Supplementary Information

Supplementary Information.
