# Supplementary material for: Over 30% efficiency bifacial 4-terminal perovskite-heterojunction silicon tandem solar cells with spectral albedo
Source: Sci Rep. 2021 Jul 30;11:15524. doi: 10.1038/s41598-021-94848-4 (PMC8324905; doi:10.1038/s41598-021-94848-4)
Supplement: Supplementary file 1 — Supplementary Information. [file 41598_2021_94848_MOESM1_ESM.pdf]

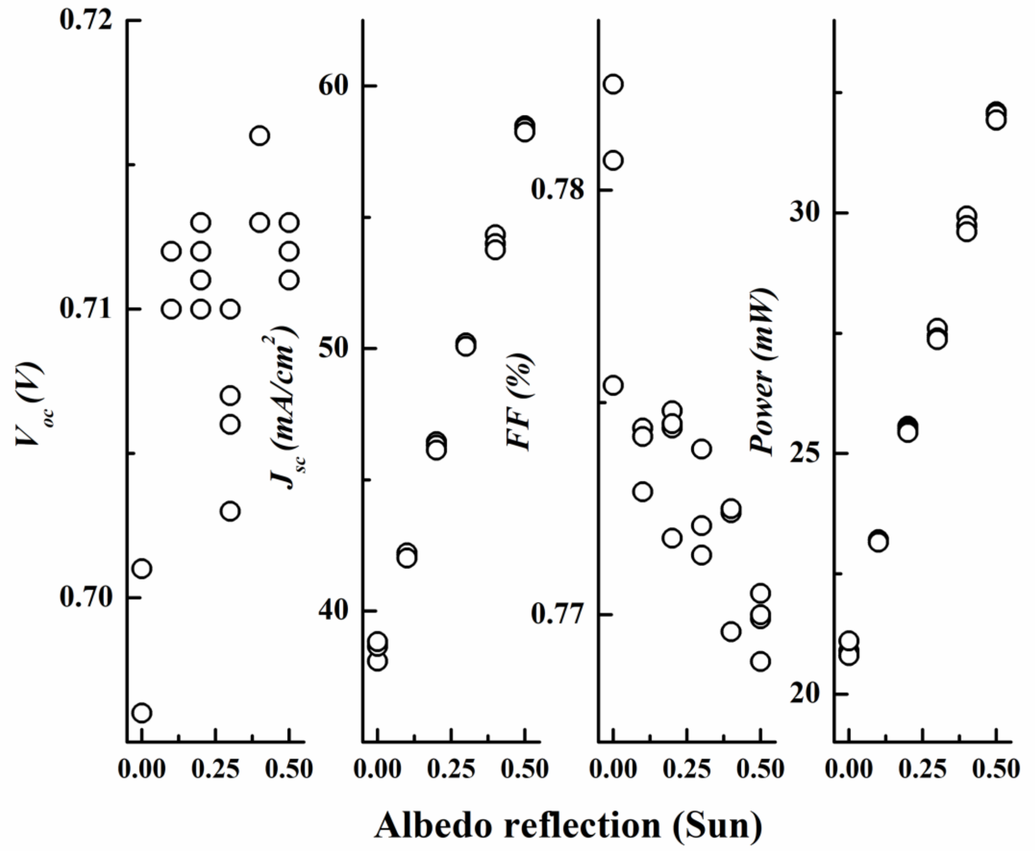

Fig. S1. V_{oc}, J_{sc} *FF* and Power of the a-Si:H/c-Si HJ solar cell without a PVK filter as a function of the albedo reflection

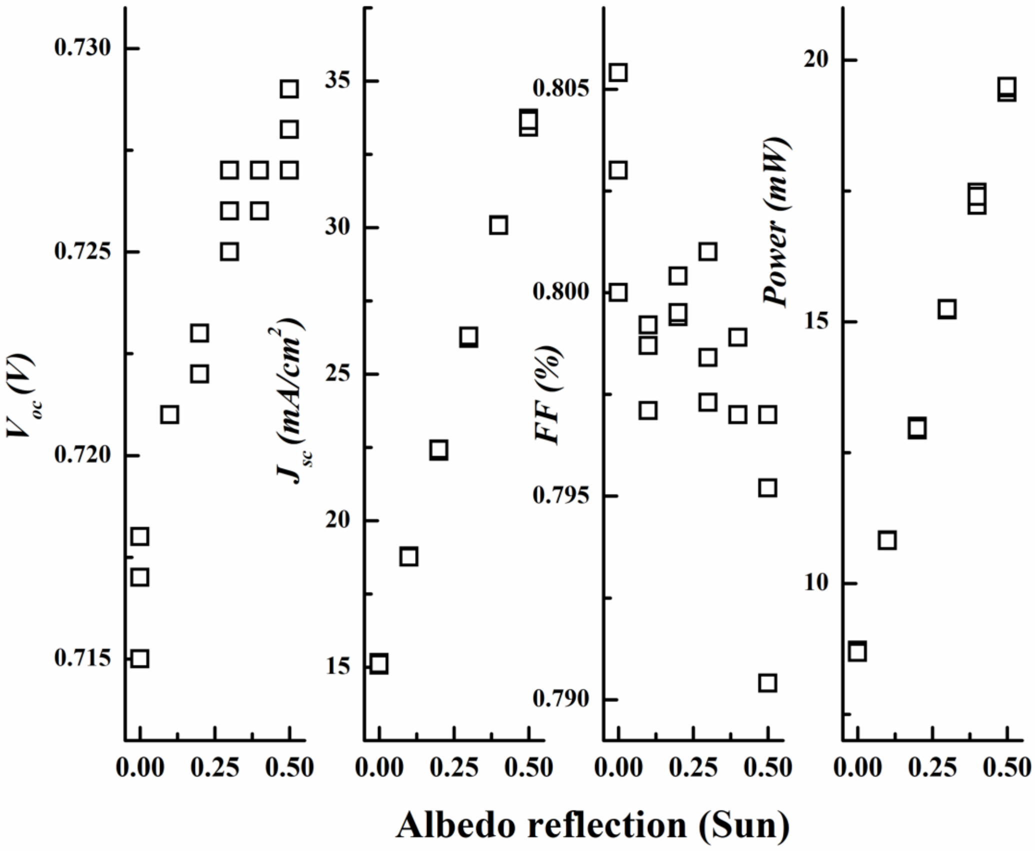

Fig. S2. V_{oc}, J_{sc} *FF* and Power of the PVK-filtered a-Si:H/c-Si HJ solar cell as a function of the albedo reflection
